# ASO Author Reflections: Enhanced Recovery in Retroperitoneal Sarcoma Surgery—From Concept to Consensus

**DOI:** 10.1245/s10434-026-19595-0

**Published:** 2026-04-13

**Authors:** Pablo Lozano Lominchar, José Manuel Asencio, Hugo Vasques

**Affiliations:** 1 Department of General Surgery and Surgical Oncology, Hospital General Universitario Gregorio Maranon, IISGM, Universidad Complutense de Madrid, Madrid, Spain; 2 Department of Surgical Oncology, Instituo Portugu고de Oncologia (IPO), Francisco Gentil de Lisboa, Lisbon, Portugal

## Past

Enhanced Recovery After Surgery (ERAS) programs have reshaped perioperative care across many surgical specialties during the past two decades. Initially developed in colorectal surgery, these pathways have progressively been adopted for increasingly complex procedures, including hepatopancreatobiliary surgery, liver transplantation, and cytoreductive surgery with hyperthermic intraperitoneal chemotherapy.^1^^,^^2^ By integrating evidence-based perioperative strategies within a multidisciplinary framework, ERAS protocols aim to attenuate surgical stress, accelerate recovery, and improve patient outcomes.

Despite this widespread adoption, the role of ERAS in primary retroperitoneal sarcoma (RPS) surgery has remained poorly defined. Surgery for RPS is among the most demanding procedures in oncologic surgery. Achieving adequate oncologic margins frequently requires extensive multivisceral resections involving adjacent organs and major vascular structures. Data from the Transatlantic Australasian Retroperitoneal Sarcoma Working Group have demonstrated the magnitude of these operations and the substantial postoperative morbidity they carry.^3^ Similarly, results from the STRASS trial highlighted the complexity of RPS surgery, with prolonged operative times and hospital stays reflecting the physiologic burden of these procedures.^4^

Until recently, perioperative management in this setting has largely depended on institutional experience rather than structured pathways. A retrospective study from Boston by Lyu et al.^5^ provided important preliminary evidence that ERAS implementation could be feasible in sarcoma surgery, demonstrating reductions in postoperative ileus, readmissions, and hospital length of stay without an increase in complications. These findings suggested that enhanced recovery strategies might offer meaningful benefits even in the context of highly complex oncologic surgery.

## Present

Building on this emerging evidence, this study sought to address a longstanding gap in the field: the lack of specific perioperative care recommendations for patients undergoing surgery for primary retroperitoneal sarcoma.

Given the rarity of RPS and the limited availability of disease-specific data, the authors adopted a multidisciplinary consensus approach involving specialists in surgical oncology, anesthesiology, medical oncology, nutrition, and perioperative medicine. Through a structured Delphi process, they developed the first set of consensus-based recommendations tailored to the perioperative management of patients undergoing RPS surgery.

These recommendations encompass the entire perioperative pathway, from preoperative patient optimization and prehabilitation to intraoperative management and postoperative recovery strategies. Although much of the available evidence originates from other areas of major abdominal surgery, these principles were carefully adapted to reflect the unique technical and physiologic challenges associated with multivisceral sarcoma resections.

Rather than prescribe rigid protocols, this consensus aimed to provide a practical framework that facilitates the implementation of enhanced recovery strategies in diverse clinical environments. In rare diseases such as RPS, in which surgical volumes are limited and practices vary widely, standardizing perioperative care may help reduce variability and support more consistent outcome assessment across institutions.

## Future

The publication of consensus recommendations represents an important step toward improving perioperative care in retroperitoneal sarcoma surgery, but it should also be viewed as the beginning of a broader effort. Future work should focus on the prospective implementation and evaluation of ERAS-based pathways specifically adapted to RPS surgery. Standardized perioperative care protocols may facilitate multicenter collaboration and enable more robust assessment of outcomes such as postoperative morbidity, recovery trajectories, and health care resource utilization.

Importantly, these pathways also provide an opportunity to explore interventions that have shown promise in other surgical settings, particularly multimodal prehabilitation programs incorporating physical training, nutritional optimization, and psychological support.^6^

As international collaboration in sarcoma research continues to expand, coordinated efforts will be essential to generate higher-level evidence and refine perioperative care strategies for this rare disease. Ultimately, integrating enhanced recovery principles into retroperitoneal sarcoma surgery not only may improve postoperative recovery, but also may contribute to a more standardized and evidence-based approach to the care of these complex patients.^7^
